# Supplementary material for: Enhanced Superconductivity in 2H-TaS2 Devices through in Situ Molecular Intercalation
Source: ACS Appl Mater Interfaces. 2024 Jul 23;16(31):41626–32. doi: 10.1021/acsami.4c04997 (PMC12865752; doi:10.1021/acsami.4c04997)
Supplement: Supplementary file 3 [file am4c04997_si_003.pdf]

Supporting Information

Enhanced Superconductivity in $2H-TaS_2$ Devices Through in-situ Molecular Intercalation

*Jose M. Pereira[1] ‡, Daniel Tezze[1] ‡, Beatriz Martín-García[1,2], Fèlix Casanova[1,2], Maider Ormaza[3], Luis E. Hueso[1,2] and Marco Gobbi[2,4] *.*

[1] CIC nanoGUNE BRTA, 20018 Donostia-San Sebastián, Spain

[2] IKERBASQUE, Basque Foundation for Science, 48013 Bilbao, Spain

[3] Departamento de Polímeros y Materiales Avanzados: Física, Química y Tecnología (UPV-EHU), 20018 San Sebastián, Spain

[4] Centro de Física de Materiales (CSIC-UPV-EHU) and Materials Physics Center (MPC), 20018 San Sebastián, Spain.

* Corresponding Author

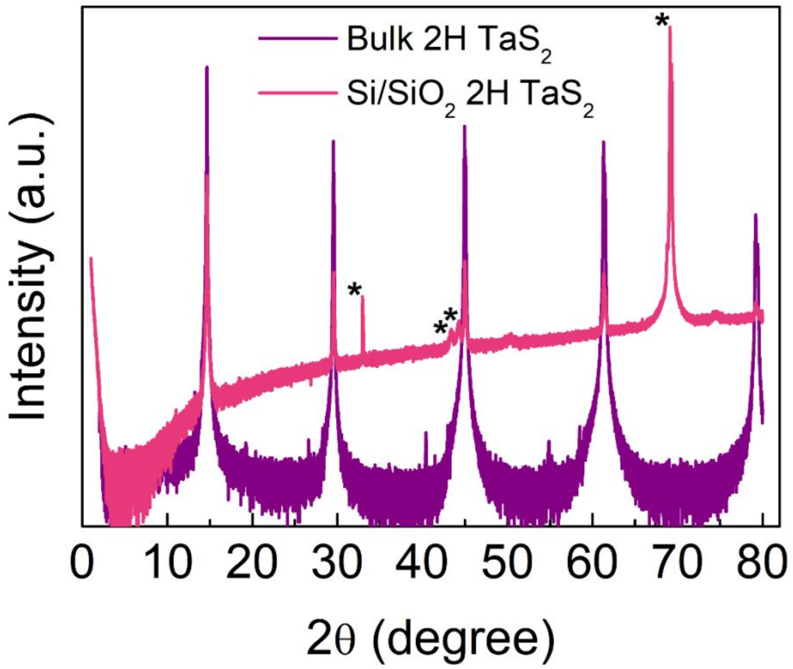

Figure S1: XRD patterns of a bulk pristine 2H TaS_2 crystal (purple line) and exfoliated 2H TaS_2 flakes on a Si/SiO_2 (300nm) substrate (pink line). Data presented in logarithmic arbitrary units scale to enhance the peak position readability as opposed to the linear scale used in Figure 1c of the original manuscript. The XRD patterns of exfoliated flakes and bulk crystal show the same peaks, indicating that the mechanical exfoliation and transfer on the substrate does not compromise the structural integrity of 2H-TaS_2. Spurious peaks originating from the Si/SiO_2 substrates are labeled with a star.

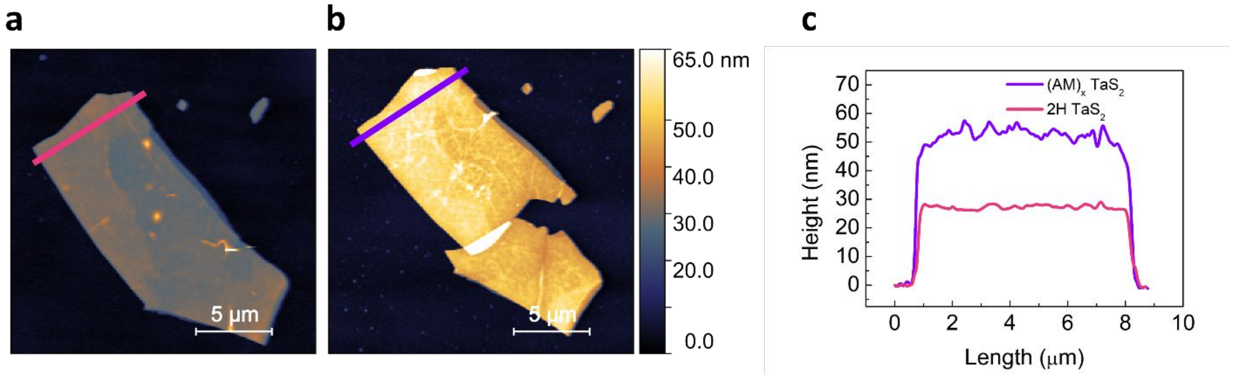

Figure S2. (a) AFM image of a pristine 2H TaS_2 flake exfoliated on a Si/SiO2 substrate. Pink line corresponds to the line taken as profile in panel c. (b) AFM image of the same flake after 30 minutes submerged in AM at room temperature and ambient conditions. Pink line and Bright purple line corresponds to the line taken as profile in panel c. (c) AFM profile comparison of the pristine 2H TaS_2 (pink line) and after intercalation with AM (bright purple line). The flake surface is notably rougher than that of the flake intercalated in AM:ACN (Fig. 3 in the main text).
